# Mimickers of novel coronavirus disease 2019 (COVID-19) on chest CT: spectrum of CT and clinical features

**DOI:** 10.1186/s13244-020-00956-6

**Published:** 2021-02-03

**Authors:** Ali H. Elmokadem, Nihal M. Batouty, Dalia Bayoumi, Basma N. Gadelhak, Rihame M. Abdel-Wahab, Mona Zaky, Sherif A. Abo-Hedibah, Ahmed Ehab, Ahmed El-Morsy

**Affiliations:** 1 Department of Radiology, Mansoura University, Elgomhoria St., Mansoura, 35516 Egypt; 2 Department of Radiology, Farwaniya Hospital, Al Farwaniyah , Kuwait; 3 Department of Radiology, Cairo University, Giza, Egypt; 4 Pulmonary Medicine Department, Mansoura University, Mansoura, Egypt; 5 Pulmonary Medicine Department, Loewenstein Lung Center, Löwenstein, Germany

**Keywords:** Computed tomography, Differential diagnosis, Coronavirus disease 2019, COVID-19, SARS-COV2

## Abstract

COVID-19 (coronavirus disease 2019) is a recently emerged pulmonary infection caused by severe acute respiratory syndrome coronavirus (SARS-CoV-2). It started in Wuhan, China, in December 2019 and led to a highly contagious disease. Since then COVID-19 continues to spread, causing exponential morbidity and mortality and threatening economies worldwide. While the primary diagnostic test for COVID-19 is the reverse transcriptase–polymerase chain reaction (RT-PCR) assay, chest CT has proven to be a diagnostic tool of high sensitivity. A variety of conditions demonstrates CT features that are difficult to differentiate from COVID-19 rendering CT to be of low specificity. Radiologists and physicians should be aware of imaging patterns of these conditions to prevent an erroneous diagnosis that could adversely influence management and patients’ outcome. Our purpose is to provide a practical review of the conditions that mimic COVID-19. A brief description of the forementioned clinical conditions with their CT features will be included.

## Key points

- Categorizing COVID-19 mimickers according to chest CT features.
- Review of clinical and chest CT characteristics of COVID-19 mimickers.
- Differentiation between COVID-19 and other mimickers.

## Background

Severe acute respiratory syndrome coronavirus-2 (SARS-CoV-2) is a new type of coronavirus that was isolated from respiratory tract samples in the city of Wuhan, Hubei Province, China, in December 2019 [1]. COVID-19 (coronavirus disease 2019) became a highly infectious disease that was officially recognized as a pandemic in March 2020 [2]. More than 49 million patients and 1.2 million deaths of COVID-19 have been reported worldwide by November 10, 2020 [3]. During the rapid spread of SARS-COV-2 globally, diagnostic methods for detecting the virus have many limitations. RT-PCR testing has low sensitivity early in the disease (ranging from 37 to 71%) [4]**.** CT chest has high sensitivity, but low specificity [4–7]. This low specificity may stem from the fact that it is difficult to distinguish COVID-19 from other diseases on chest CT [8].

In order to optimize patient management, medical care and disease control, radiologists and chest physicians should be aware of CT chest features that distinguish COVID-19 from other etiologies [9]. The aim of the review is to differentiate between the COVID-19 findings on CT and other mimickers.

## COVID-19

Patients infected with COVID-19 usually presents with fever (88.7–98%), cough (67.8–76%) and dyspnea (55%), less commonly headache, fatigue, hemoptysis conjunctival congestion and loss of smell [10, 11]. Abdominal symptoms such as nausea, vomiting and diarrhea are possible [11]. The disease can progress to acute respiratory distress syndrome, metabolic acidosis, septic shock, coagulation dysfunction and multi-organ failure [12]. Laboratory findings in COVID-19 are a decreased lymphocyte count and an increased CRP and high-sensitivity C-reactive protein level [10–12]. Lymphopenia was reported to be a dependable indicator to categorize the moderate, severe, and critical ill types [13].

COVID-19 patients are classified as having minimal, common, severe and critical illness [12]. Minimal disease patients have subtle clinical symptoms. Common cases have symptoms such as fever and mild coughing. Severe cases are the ones meet any of the following criteria: (1) resting blood oxygen saturation ≤ 93%; (2) respiratory rate ≥ 30 beats/min; or (3) oxygen concentration ≤ 300 mmHg. Critical patients can be affected with one of the following: (1) respiratory failure needing mechanical ventilators; (2) shock; and (3) organ failure requiring intensive care management.

**Fig. 1:**
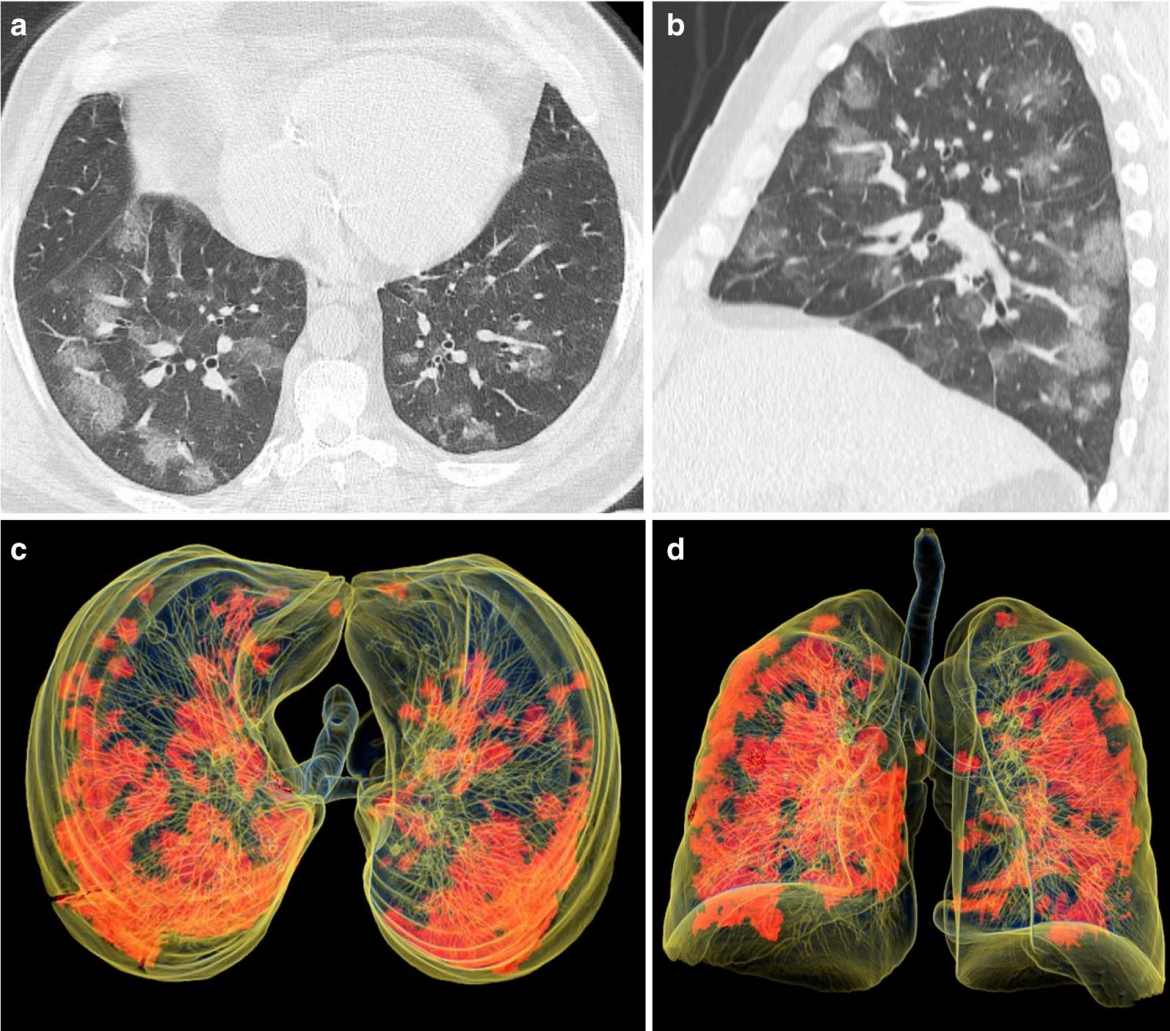
Typical CT imaging features for COVID-19. **a**, **b** A 54-year-old man with positive RT-PCR test, axial and sagittal reformatted CT images shows multifocal rounded GGO with predominant peripheral distribution. **c**, **d** Three-dimensional semitransparent volume-rendered reconstructions show the peripheral and basal distribution of the ground-glass opacities across both lungs

**Fig. 2:**
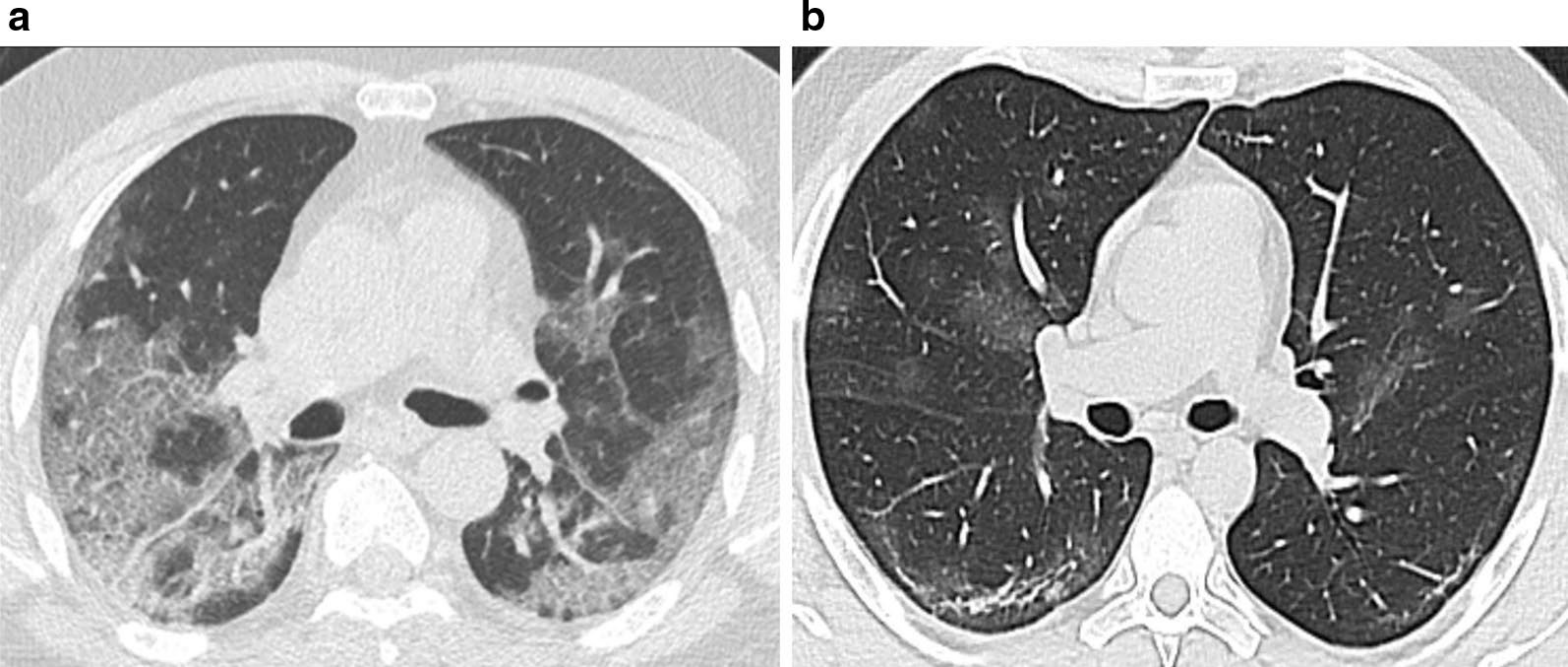
Typical CT imaging features for COVID-19. **a** A 72-year-old woman with positive RT-PCR test, axial CT image shows multifocal peripheral GGO with superimposed interlobular septal thickening and visible intralobular lines (“crazy-paving”). **b** A 46-year-old man with a positive RT-PCR, axial CT image shows bilateral multifocal rounded and peripheral GGO with bilateral posterior subpleural bands

**Fig. 3:**
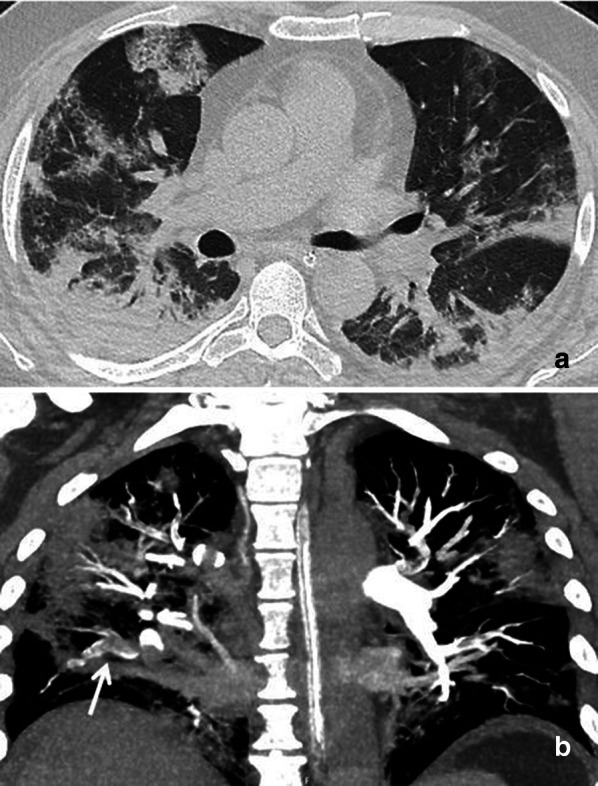
A 50-year-old man with a positive RT-PCR. **a** Axial CT image shows moderate COVID-19 pneumonia manifested by peripherally located GGO and consolidations. **b** Coronal pulmonary CT angiogram shows multiple filling defects in segmental and subsegmental pulmonary artery branches, clearly seen in the right inferior pulmonary branches (white arrow)

Ground-glass (GGO) pattern with or without consolidation is the most common feature of COVID-19 infections. They are usually bilateral, multifocal and peripheral with posterior or lower lung zone distribution [6, 7, 14–16]. Ground-glass opacification is defined as hazy increased lung opacity with preserved bronchovascular margins contrary to consolidation that is defined as a homogeneously increased parenchymal attenuation with obscuration of margins of airway and vessel walls [14]. A different unifocal pattern of GGO was reported by Zhou, et al. in early phase of disease, commonly at the inferior lobe of right lung [17]. Furthermore, Bernheim, et al. reported a significant number of cases having opacities of non-specific distribution and non-predominant perihilar pattern [6]. GGO has also been frequently reported to have a “crazy paving” pattern [6, 7, 14, 18] that is defined as thickening of interlobular septa and intralobular lines with superimposed GGO [17, 19]. Vascular dilatation and traction bronchiectasis are typical findings found in the GGO detected in COVID-19 patients [20]. Architectural distortion with the formation of subpleural bands was reported in some cases during a peak stage of the disease [9]. Pulmonary embolism was found among patients with COVID-19 and was linked to elevated D-dimer [21]. Other findings typically were seen in infection as thickening of bronchial wall, mucoid impactions and centrilobular nodules (tree-in-bud), and lymphadenopathy and pleural effusion are rarely observed [6, 14]. Figures 1, 2 and 3, and Additional file 1 demonstrate typical CT features of COVID-19 pneumonia.

CT Findings in patients with COVID-19 vary according to the timing of imaging. A negative CT during first two days after onset of symptoms with GGO usually develops between first and fourth days after symptom onset with the peak at 6–13 days have been reported [6, 9, 18, 22]. The frequency of consolidation, mixed patterns and reversed halo increases later in the disease [6, 9, 18].

The Radiological Society of North America expert consensus proposed four categories to report chest CT findings attributed to COVID-19 (typical, indeterminate, atypical and negative for pneumonia) [11]. Based on this classification, we categorized the mimics into three categories (Table 1): The first and second category based only on similarity to the imaging features of COVID-19 regardless of the differences in clinical course and the third category based on similarity to COVID-19-related clinical manifestations.

1. CT mimickers of typical COVID-19 that displays bilateral, peripheral GGO or multifocal rounded GGO of rounded morphology with or without consolidation, “crazy-paving” pattern or reversed halo sign.
2. CT mimickers of indeterminate COVID-19 that manifests as multifocal, diffuse, perihilar or unilateral GGO with or without consolidation, non-specific distribution or non-rounded GGO.
3. Conditions presented by clinical scenarios that mimic COVID-19 but with atypical CT features such as lobar or segmental consolidation without GGO, pulmonary nodules (centrilobular or “tree in-bud”), pulmonary cavitation, smooth interlobular septal thickening, pleural effusion and lymphadenopathy. Table 2 summarizes the CT findings, extra-pulmonary manifestation and laboratory findings of COVID-19 and mimickers.

**Table 1:** Proposed reporting categories for CT findings related to COVID-19 [11]

COVID-19 pneumonia Imaging classification	CT findings	Differential diagnosis
CT mimickers of typical COVID-19 Commonly reported imaging features of greater specificity for COVID-19 pneumonia	Typical findings are: 1. Peripheral, bilateral, GGO with or without consolidation or crazy-paving 2. Multifocal GGO of rounded morphology with or without consolidation or crazy-paving 3. Reverse halo sign or other findings of organizing pneumonia	1. Influenza pneumonia 2. SARS and MERS pneumonia 3. Organizing pneumonia 4. Connective tissue disease (RA) 5. Drug toxicity 6. Acute interstitial pneumonia
CT mimickers of indeterminate COVID-19 Commonly reported imaging features of greater specificity for COVID-19 pneumonia	Absence of typical features AND presence of: 1. Multifocal, diffuse, perihilar or unilateral GGO with or without consolidation 2. Lacking of specific distribution 3. Non-rounded or non-peripheral GGO	1. Other causes of viral pneumonia 2. Atypical bacterial infections 3. Pneumocystis infection 4. Pulmonary edema 5. ARDS 6. Acute hypersensitivity pneumonitis, 7. Eosinophilic pneumonia 8. Diffuse alveolar hemorrhage 9. Pulmonary alveolar proteinosis
Conditions presented by clinical scenarios that mimic COVID-19 but with atypical CT features Uncommonly or not reported CT features	Absence of typical or indeterminate features AND presence of: 1. Isolated lobar or segmental consolidation without GGO 2. Discrete small nodules (centrilobular or “tree in-bud”) 3. Lung cavitation 4. Smooth interlobular septal thickening with pleural effusion	Alternative diagnoses rather than COVID-19 should be considered such as typical bacterial pneumonia, TB or other non-infective processes

**Table 2:** CT findings, extra-pulmonary manifestation and characteristic laboratory findings of COVID-19 and its mimics

Disease			CT findings							Extra-pulmonary manifestation	Characteristic Laboratory findings
			Location	GGO	Consolidation	Crazy paving	Nodules	Pleural effusion	Other findings		
(1) COVID-19			Multi-/(less) Unifocal Bilateral Peripheral Posterior or lower zonal	++++	± (more later in the course)	++	(R) (either Tree-in-bud or centrilobular)	(R)	(T) Vascular dilation and traction bronchiectasis (S)Architectural distortion and subpleural bands (R) bronchial wall thickening, mucoid impaction or LNs	Abdominal symptoms Acute necrotizing encephalopathy Myocarditis Acute kidney injury	(- -) Lymphocyte count (++) CRP
(2) Viral pneumonia		(a) Influenza A, B and C	Focal Multifocal Diffuse	++++	++		++ (Centrilobular)	++	Pseudo-cavitation Pneumatocele LNs ± ARDS		Lymphocytosis or lymphopenia
		(b) Coronaviridae (SARS/MERS)	Upper and lower resp. infection Multifocal	++++	++			(R)	(C) Reticulation (R) Cavitation (R) LNs		
		(c) HPIV	Multifocal	++	++++ (patchy)		+ (Centrilobular)		Bronchial wall thickening		
		(d) Human adenovirus	Bilateral Multifocal	+++	Patchy (lobar/segmental)		++++ (Centrilobular)	++++			
		(e) Herpes viruses	Multifocal segmental Diffuse	++++	+		(C) Multiple hemorrhagic	++++	(C) fungal pneumonia	Gingivostomatitis, pharyngitis and herpes labialis (HSV)	
		(f) Human bocavirus	Diffuse Along bronchovascular bundles	++++	++++ (patchy)	++++					
		(g) Rhinovirus	Multifocal	++	+		(R)	(R)			
		(h) RSV	Airway centric		±		++++		Bronchial wall thickening		
		(j) Measles	Peribronchial				Nodular/reticulo-nodular infiltrates	+++	Thick interlobular septae (S) Fibrosis (C) Hilar LNs	Lymphadenopathy gastroenteritis, Encephalitis	
(3) Atypical Bacterial pneumonia (Mycoplasma P. and Chlamydia P.)			Uni-/Bilateral	+	+		+ (Centrilobular/peribronchovascular)	(R)	Thick bronchovascular bundle (R) atelectasis Chlamydia P. reticular or linear opacity, airway dilatation and emphysema	Fatigue, Malaise	Mildly ( +) or normal WBC
(4) Pneumocystis jiroveci pneumonia (PJP)			Central with peripheral sparing (++) in upper lobes	++++ (extensive in non-HIV)	+++ (in non-HIV patients)	++ (in advanced cases)	+ (solitary/multiple)		(C) pulmonary cysts spontaneous pneumothorax (R) cancer-like mass (S) interstitial fibrosis	Tachypnea, tachycardia and cyanosis	(++) LDH (not specific)
(5) Pulmonary edema cardiogenic/non-cardiogenic (ARDS)			Bilateral Perihilar symmetrical (cardiogenic)	++++	+++ in ARDS (heterogeneous, peripherally and dependent)	++		+++ in cardiogenic edema	(T) Anteroposterior density gradient Pulmonary cysts lately	Cardiomegaly Cyanosis, dyspnea tachypnea in cardiogenic edema	
(6) Hypersensitivity pneumonitis			**Acute:** normal or diffuse **Subacute:** mid/upper lung zone **Chronic:** peribronchovascular (++) at mid/upper lung zones	+++			**Acute:** centrilobular ground-glass nodules **Subacute:** poorly defined CL nodules		**Subacute:** air trapping (S) Thin walled cysts **Chronic:** fibrotic changes (septal thickening, traction bronchiectasis, honey combing)	Cyanosis, fatigue, anorexia and weight loss	Non-specific increased ESR and CRP
(7) Diffuse alveolar hemorrhage			Diffuse Patchy Lobular Perihilar Gravity-dependent	+++	+++	±				Hemoptysis, anemia, signs of collagen-vascular disease	Thrombocytopenia Coagulopathy
(8) Pulmonary alveolar proteinosis			Patchy Subpleural sparing Geographic distribution ++in lower lung			+++ (T)	No	No	No LNs	Weight loss	Elevated LDH
(9) Eosinophilic P.			Bilateral non-segmental areas Middle and upper lobar predominance	+	++++ (no subpleural spacing)		+	+	(S) lung reticulations and mediastinal LNs		Alveolar and blood eosinophilia ++IgE ++ ESR Peripheral thrombocytosis
(10) Interstitial lung disease	(A) COP		Multifocal Subpleural Peribronchial distribution	++++	++++		+		(R) masses with regional architectural distortion and interlobular septal thickening. (T) “atoll sign”	Weight loss, generalized bone aches	
	(B) DAD/AIP		Symmetric and bilateral Lower lobe predominance Sparing C/P recesses	++++	++ (in the dependent portions)	+			Later fibrotic phase of DAD Architectural distortion, honeycombing and traction bronchiectasis (similar to UIP)		
	(C) CTD associated P.		Subpleural and basal predominance	(S) extensive	(S) Segmental		(S)		(C) reticular densities, traction bronchiectasis, honey combing and clustered cysts (S) mosaic perfusion, discrete cysts and air trapping	Systemic manifestation according to type of disease	Detected antinuclear antibodies (ANA) and an antibody to ribonucleoprotein (RNP)

(C): common, (R): rare, (S):some cases, (T): typical

## Chest CT mimickers of typical COVID-19

### Viral pneumonias

Viruses are considered the most common pathogens causing acute respiratory tract infections [23] usually, the clinical signs and symptoms of viral pneumonia are non-specific, and the clinical course vary according to patient age and immune status [24]. CT features of viral pneumonia are variable and may be affected by immunity and pathophysiology of the virus [23]. Differentiating between COVID-19 and non-COVID19 pneumonia is a crucial need in the current situation to reduce unnecessary quarantine for suspected patients. Bai et al. [9] demonstrate that radiologists are capable of distinguishing COVID-19 from other viral etiologies of pneumonia on chest CT with high specificity (93–100%).

*HPIV (human parainfluenza virus)* presents on CT as multifocal patchy consolidation with GGO of non-specific distribution and in one-fourth of patients show centrilobular nodules with bronchial wall thickening [25].

**Fig. 4:**
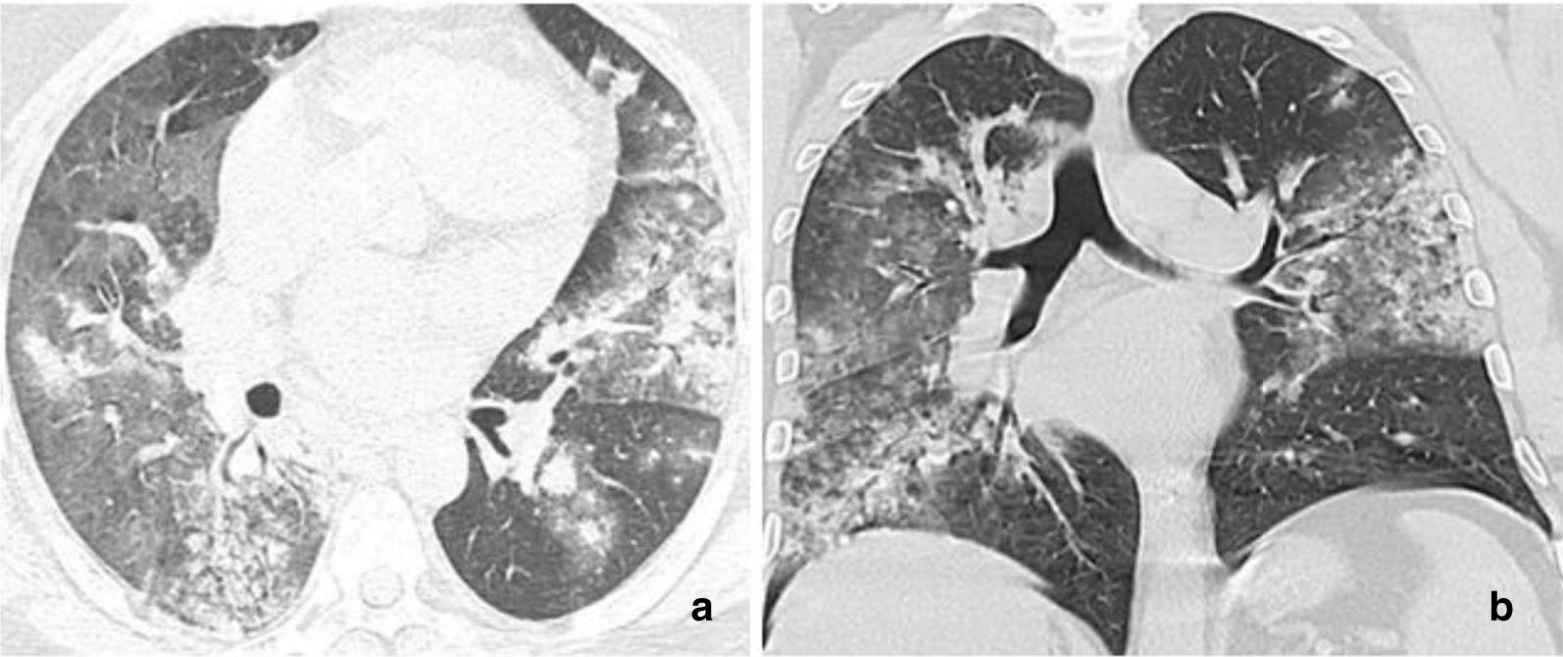
Viral infection mimicking typical COVID-19. **a**, **b** Axial and coronal reformatted CT image obtained for a 52-year-old man with positive PCR test for influenza (**a**) pneumonia show diffuse and multifocal GGO with peripheral consolidation and centrilobular nodules scattered in both lungs

*Influenza A, B and C pneumonia* (Fig. 4) manifested on CT as focal, multifocal, or diffuse GGO with areas of consolidation. Unlike COVID-19, centrilobular nodules, pseudocavitation, pneumatocele formation and lymphadenopathy are commonly seen. Pleural effusion and cavitation can develop later in the course of the disease [23, 24]. An H1N1 (a subtype of Influenza A) pandemic reported in more than 70 countries with 30,000 cases of infection occurred in 2009 [26].

*Corona virus* cause pneumonia, bronchiolitis and even acute respiratory distress syndrome (ARDS). SARS Coronavirus (severe acute respiratory syndrome) outbreak originated from Guangdong Province, China, occurred during 2002–2003 [27]. In 2012, another coronavirus-related epidemic occurred in the Middle East, identified as MERS (Middle East respiratory syndrome) [27]. CT features of both diseases are similar to COVID-19 pneumonia including multifocal GGOs with less common consolidations. Reticulation is noted after the second week. Cavitation, lymphadenopathy or pleural effusion are not common findings [23].

### Organizing pneumonias (OP)

**Fig. 5:**
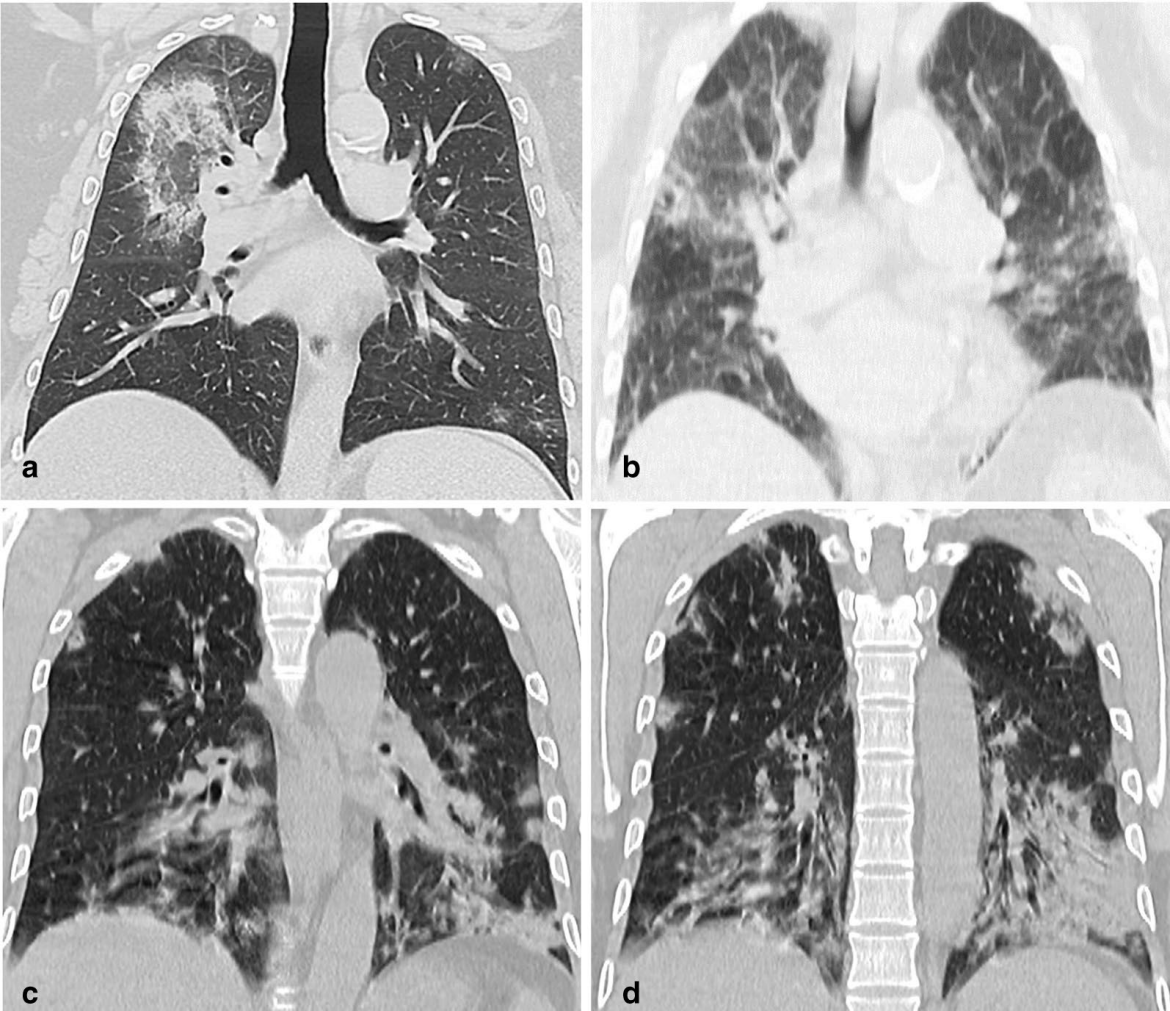
Organizing pneumonia mimicking typical COVID-19. **a** Cryptogenic organizing pneumonia in a 55-year-old man with history of chest infection not responding to multiple courses of antibiotics, coronal reformatted CT image shows reversed halo sign in the right lung with small areas of GGO in the left lung. Transbronchial biopsy showed findings of COP. **b** Rheumatoid arthritis induced interstitial pneumonia in a 67-year-old woman, coronal reformatted CT image shows bilateral ground-glass opacities with interlobular and interstitial thickening. **c**, **d** Cyclophosphamide-induced OP in a 56-year-old man with Hodgkin disease, coronal reformatted CT images show peripheral multifocal areas of poorly defined focal consolidation, small areas of GGO and bronchial wall thickening

*Cryptogenic organizing pneumonia (COP)* is a disease of unknown etiology previously named bronchiolitis obliterans organizing pneumonia (BOOP). It is commonest among the 55–60 age groups and it usually presents with a several-month history of nonproductive cough, low-grade fever, malaise and shortness of breath in contrary to the rapidly progressive course of COVID-19. COP can be caused by different infective and non-infective causes and results in bronchiolar occlusion [28]. On CT, COP usually shows multifocal GOO or consolidations predominantly subpleural and peribronchial distribution. Less commonly, CT includes variable-sized solid nodules with peribronchial or peribronchiolar distribution with architectural distortion and interlobular septal thickening [29]. COP is characterized by areas of clearing consolidation with central ground-glass density, which are known as the reversed halo sign or "atoll sign" (Fig. 5); however, these signs are only seen in less than 20% of patients [28, 29]. Reversed halo sign can also be secondary to other causes such as pulmonary mucormycosis, invasive pulmonary aspergillosis, pulmonary infarction due to venous thromboembolism, granulomatosis with polyangiitis, sarcoidosis, lymphomatoid granulomatosis and lipoid pneumonitis. It is uncommon feature for COVID-19 but possible finding that occurs later with disease progression.

### Connective tissue disease (CTD)-associated pneumonias:

Rheumatoid arthritis (RA), systemic sclerosis, scleroderma, polymyositis or dermatomyositis, systemic lupus erythematosus and Sjögren’s syndrome can all be associated with the development of interstitial lung disease. RA is the commonest among the aforementioned disease and is characterized by subacute or chronic inflammatory polyarthropathy. Parenchymal affection in rheumatoid patients has two common types, usual interstitial pneumonia (UIP) pattern and non-specific interstitial pneumonia (NSIP) pattern [30]. Other less common patterns may happen as OP and obliterative bronchiolitis that may simulate COVID-19 on chest CT; however, the clinical course is different considering the gradual progressive course of CTD-associated pneumonias. The overlap between it manifests on CT by extensive GGO and patchy mosaic perfusion (Fig. 5), solid nodules, discrete cysts, air trapping and segmental consolidations [31].

NSIP occurs more commonly in association with other connective tissue diseases such as scleroderma and polymyositis or dermatomyositis interstitial lung disease (PM/DM ILD)**.** The most characteristic CT finding in NSIP is GGO, which is usually bilateral and symmetric with lower lobe predominance. Other common CT findings include traction bronchiectasis, fine reticulation and volume loss of lower lobes. Unlike COVID-19, subpleural sparing of the posterior regions of the lower lobes and mediastinal lymph node enlargement are relatively common features in NSIP [31]. Furthermore, consolidation is not a common finding in NSIP and only seen when it is superimposed with organizing pneumonia.

### Drug induced acute lung injury

Drug toxicity is a common and underdiagnosed cause of acute lung disease. Radiologic features and clinical symptoms of drug-induced lung injury differ according to the histopathological pattern of affection. The histopathologic manifestations that could simulate COVID-19 disease include diffuse alveolar damage (DAD) and OP.

Bleomycin, busulfan, carmustine, cyclophosphamide, mitomycin, melphalan and gold salts are the most common drugs that cause DAD [32], while drugs that most commonly induce OP are bleomycin, gold salts, methotrexate and cyclophosphamide (Fig. 5) [32, 33]. Recently, a crucial side effect has arisen as a lung-specific toxicity caused by an epidermal growth factor receptor (EGFR) tyrosine kinase inhibitors (TKIs) for the treatment of non-small cell lung cancer (NSCLC) [33]. Fibrosis can regress significantly, remain stable, or evolve to honeycomb lung based on the severity of the lung injury [34].

### Diffuse alveolar damage (DAD)/acute interstitial pneumonia (AIP)

**Fig. 6:**
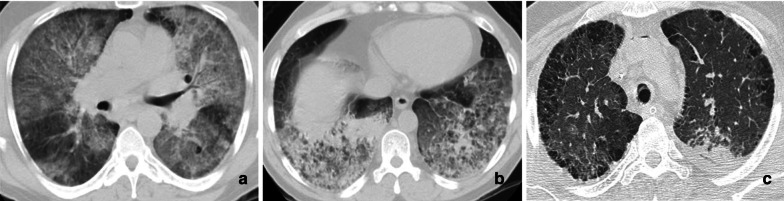
Acute interstitial pneumonia mimicking typical COVID-19 in a 48-year-old man presented with symptoms simulating ARDS. **a**, **b** axial CT images show bilateral extensive diffuse GGO and few areas of consolidation with interlobular and intralobular septal thickening, and mild bronchial dilatation. Transbronchial biopsy showed features of diffuse alveolar damage. **c** Follow-up axial CT image shows after one month shows subpleural honeycombing, fibrotic bands and traction bronchiectasis

Acute interstitial pneumonia is rapidly progressive pneumonia that occurs in an otherwise healthy person over a few days to weeks [31]. It is indistinguishable both clinically and histologically from ARDS. AIP occurs in wide age range (mean age 50 years) [35]. CT findings of AIP are similar to those of ARDS and the sever form of COVID-19, though AIP more often produces symmetric and bilateral GGO more commonly in the lower lobes with the tendency to spare the costophrenic recesses crazy paving pattern is occasionally present. Areas of consolidation predominantly located in the dependent portions but less extensive than GOO. The delayed fibrotic phase of DAD simulates UIP (Fig. 6), and it is associated with architectural distortion, traction bronchiectasis and honeycombing [31, 35].

## Chest CT mimickers of indeterminate COVID-19

### Viral pneumonias

**Fig. 7:**
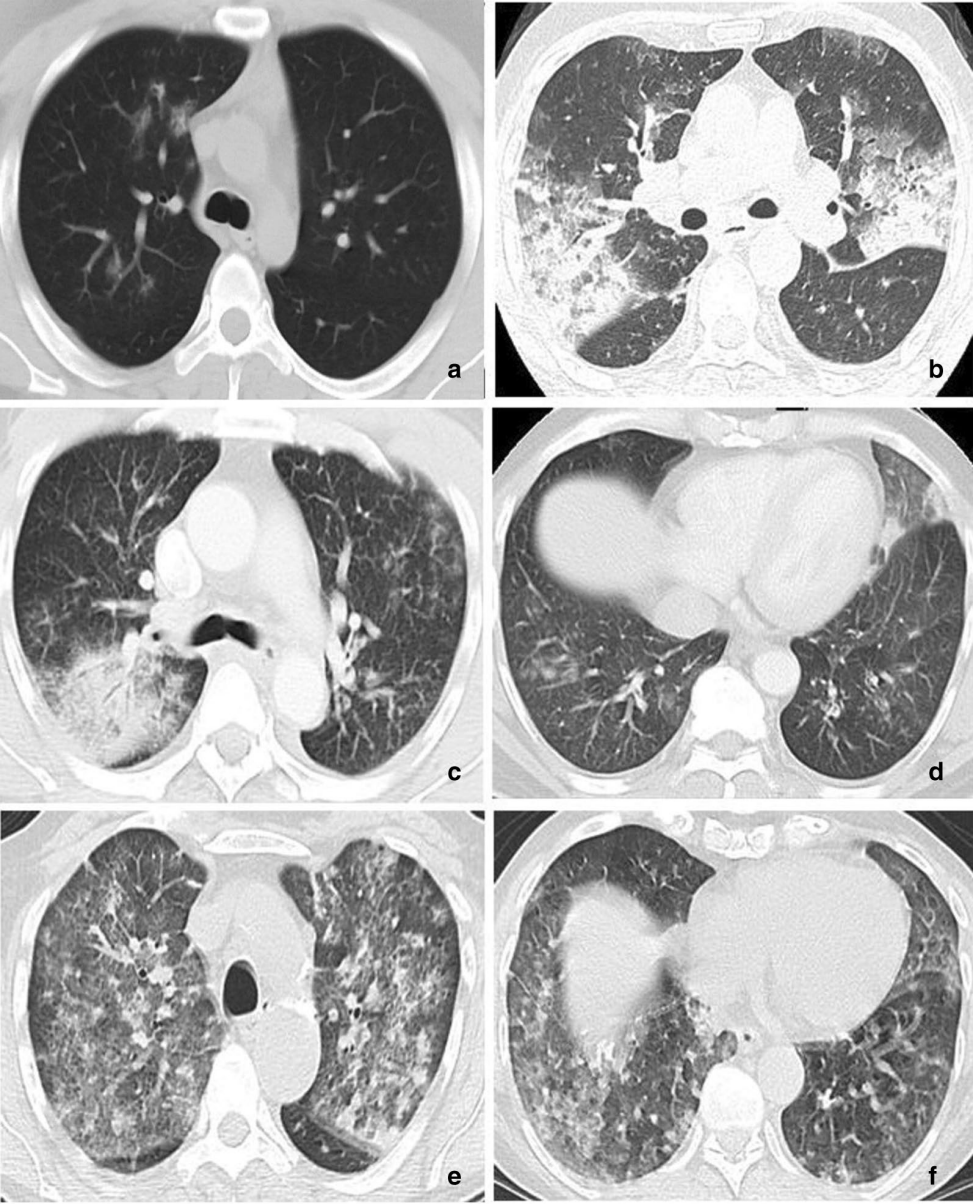
Indeterminate COVID-19 and viral pneumonia mimickers. **a**, **b** Indeterminate CT features for COVID-19 in two different patients with positive RT-PCR test, axial CT images show unilateral small areas of non-peripheral GGO (**a**) and bilateral multifocal non-rounded GGO of non-specific distribution (**b**). **c**, **d** Adenovirus pneumonia in a 48-year-old man, axial CT images show bilateral area of consolidation with nearby GGO and bilateral centrilobular nodules. **e**, **f** HSV pneumonia in a 75-year-old woman, axial CT images show widespread bilateral GGO of non-rounded morphology, small areas of consolidation and diffuse interlobular septal thickening

Viral pneumonia may have CT features mimicking indeterminate COVID-19 (Fig. 7) including perihilar distribution, centrilobular/tree in pud nodules, pleural thickening, effusion or lymphadenopathy.

*Human Adenovirus* pneumonia shows bilateral multifocal GGO with patchy consolidations and may show lobar or segmental distribution unlike COVID-19**.** Centrilobular nodules and pleural effusion are common [23].

*Herpes viruses* include HSV types 1 and 2 and CMV. A predominantly multifocal segmental or diffuse GGO is a common feature of herpes pneumonia, consolidation is less evident [24]. The presence of multiple hemorrhagic nodules, associated fungal pneumonia or pleural effusion is frequent with HSV infection [23, 25].

*Human bocavirus* manifests as diffuse GGO or patchy consolidation along bronchovascular bundles associated with interlobular septal thickening [23].

*Rhinovirus A, B and C* severe pneumonia characterized by bilateral patchy consolidations with multifocal GOO and interlobular septal thickening [36]

### Atypical bacterial pneumonias

**Fig. 8:**
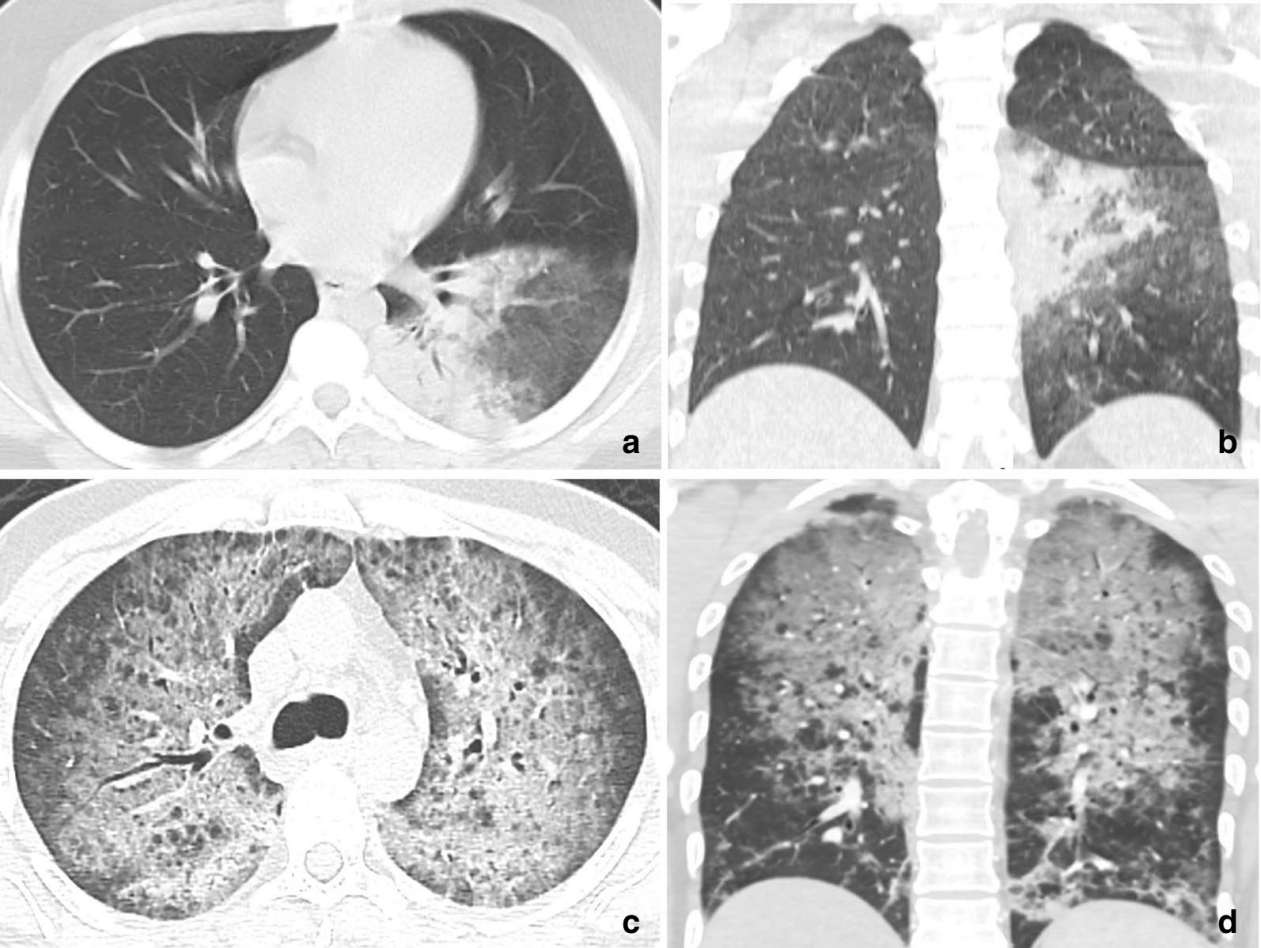
Atypical infection mimicking indeterminate COVID-19 and viral pneumonia mimickers. **a**, **b** A 39-year-old man with Mycoplasma pneumonia, axial and coronal reformatted CT images shows unilateral lobar consolidation with nearby GGO and centrilobular nodules. **c**, **d** Pneumocystis jirovecii pneumonia in a 36-year-old man with positive HIV test, axial and coronal reformatted CT images show extensive bilateral ground-glass opacities with relative subpleural sparing

Mycoplasma (M) pneumonia and chlamydia (C) pneumonia are known as atypical pneumonia, patients present with dry or productive cough, fatigue and malaise with mildly elevated or normal white blood cell count [37, 38]. Both C pneumoniae pneumonia and M pneumonia (Fig. 8) cause infectious bronchiolitis or bronchopneumonia that may simulate COVID-19 as they are presented with unilateral or bilateral areas of consolidation or GGO and thickened broncovascular bundle [37]. However, the presence of centrilobular or peribronchovascular nodules may help in differentiation from COVID-19. Pleural effusion and atelectasis are less frequent features. C pneumonia had a higher frequency of reticular or linear opacity, airway dilatation and pulmonary emphysema that represent its chronic nature [38].

### Pneumocystis jirovecii pneumonia (PJP)

Pneumocystis jirovecii is an atypical fungus that causes pneumonia in immunocompromised patients [39]. The presentation of PJP in a patient with HIV infection typically is subacute, characterized by a gradual onset (over 1 month duration) of dry cough and dyspnea with signs of respiratory compromise, including tachycardia, tachypnea and cyanosis [40]. On CT, extensive GGO is the principal finding in PJP (Fig. 8). In HIV-related PJP, CT shows commonly central distribution of GGO with relative peripheral sparing and predilection for the upper lobes. CT in non-HIV patients shows a greater extent of GGO with more common and rapidly developing lung consolidation [39, 40]. In advanced disease, crazy paving pattern and consolidation are noted. Interstitial fibrosis may occur in patients recovering from PJP, thus known as chronic Pneumocystis pneumonia [39].

### Pulmonary edema

**Fig. 9:**
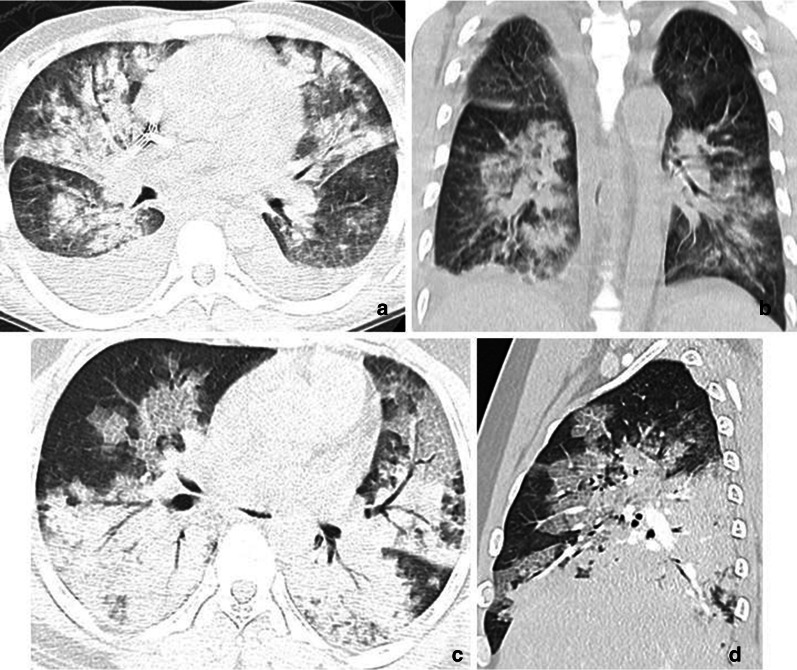
Non-infectious mimickers of indeterminate COVID-19. **a**, **b** A 73-year-old man with pulmonary edema, axial and coronal reformatted CT images show bilateral perihilar consolidation and to less extent GGO associated with bilateral pleural effusion and pericardial effusion in mediastinal window (not shown). **c**, **d** A 58-year-old woman with acute respiratory distress syndrome, axial and sagittal reformatted CT images shows dependent consolidation and multifocal non-rounded GOO superimposed with interlobular septal thickening (crazy paving)

Pulmonary edema has various types and etiologies with different radiological patterns: cardiogenic pulmonary edema (e.g., congestive heart failure), non-cardiogenic pulmonary edema and fluid overload (e.g., renal failure) [41]. Both pulmonary edema and COVID 19 infection may have bilateral GGO but with different distribution and other associated signs. The diffuse ground-glass pattern of cardiogenic pulmonary edema tends to be perihilar, bilateral and symmetrical (Fig. 9). Cardiogenic pulmonary edema is presented by acute dyspnea, associated with cardiomegaly and pleural effusion is common, while ARDS has dense-dependent consolidations, with or without interlobular septal thickening, and pleural effusion may occur [41, 42]. Pulmonary edema can also be presented by crazy paving pattern [42].

### Acute respiratory distress syndrome (ARDS)

ARDS in adults is a non-specific catastrophic response of the lung to injury. Pneumonia, aspiration of gastric contents, toxic fumes inhalation and near drowning are the common pulmonary causes. The extra-pulmonic causes are systemic sepsis, non-cardiogenic shock, severe trauma, pancreatitis, drug overdose and multiple blood transfusions [43]. Signs and symptoms are dyspnea, cyanosis, tachypnea and hypoxemia mimicking those of cardiogenic pulmonary edema. Similar to COVID-19, CT features in extra-pulmonary ARDS are bilateral GGO and consolidations distributed peripherally and mainly in the dependent parts of the lung (Fig. 9) [42]. In pulmonary ARDS, the distribution of lung changes is more likely to be asymmetrical, with mixed GGO and non-dependent consolidation [43]. Pleural effusion and bronchial dilatation within GGO are equally common to both types of ARDS.

### Hypersensitivity pneumonia (HP)

**Fig. 10:**
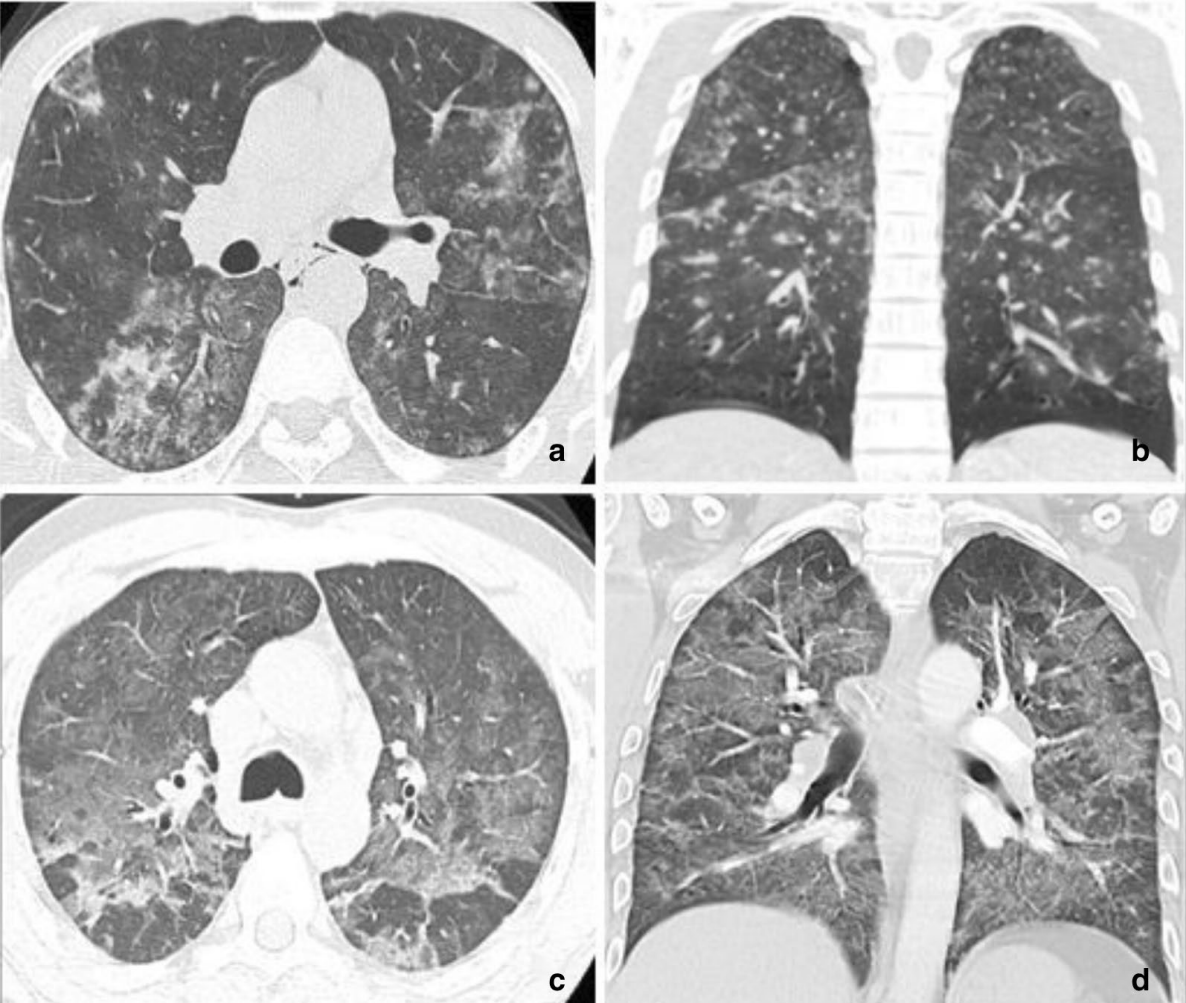
Non-infectious mimickers of indeterminate COVID-19. **a**, **b** A 33-year-old bird breeder with subacute hypersensitivity pneumonitis, axial and coronal reformatted CT images shows patchy or diffuse bilateral ground-glass opacities associated with poorly defined centrilobular nodules and lobular areas of reduced attenuation indicative of air trapping. **c**, **d** A 38-year-old man with acute eosinophilic pneumonia, axial and coronal reformatted CT images shows multifocal non-rounded GOO associated with inter- and intralobular septal thickening and thickened bronchovascular bundles

HP is a diffuse parenchymal lung disease caused by inhalation and sensitization to a long list of aerosolized antigens [44]. HP is often categorized into acute, subacute and chronic stages. Acute HP symptoms range from dyspnea, cough and myalgia to extensive pulmonary edema, while subacute HP symptoms are lesser and more insidious and may be associated with cyanosis, fatigue and weight loss [44, 45]. CT in acute HP may be normal or show diffuse ground-glass opacity. In subacute HP, CT often shows GGO similar to COVID-19 though the presence of faint centrilobular nodules and air trapping more in the upper and middle lung zones are characteristic features for subacute HP that help in differentiation between both diseases (Fig. 10). Lobular areas of reduced attenuation are often seen in expiratory phase and indicative of air trapping. Sometimes thin-walled cysts (< 15 mm in diameter) may be found. Additionally, the head-cheese sign is relatively specific for HP and consists of a constellation of GGO, air trapping and normal intervening lung with geographic margins [45]. In chronic HP, fibrotic changes as traction bronchiectasis and honeycombing are found [31, 45].

### Eosinophilic pneumonia

An idiopathic disease that usually affects middle age with relative predominance in females and asthmatic patients [46]. Diagnosis of eosinophilic pneumonia is complex and usually based on the association of the respiratory and general manifestations (including shortness of breath, dyspnea and fever) with characteristic laboratory findings as alveolar and blood eosinophilia. In CT, there are typical bilateral non-segmental areas of airspace consolidation (no subpleural sparing) with predominance for the middle and upper lobes (Fig. 10), unlike the lower lobe predominance seen in COVID-19. Less common presentations include GGO, lung reticulations and solid pulmonary nodules. Pleural effusion and mediastinal lymphadenopathy may be seen [42, 46].

### Diffuse alveolar hemorrhage (DAH)

**Fig. 11:**
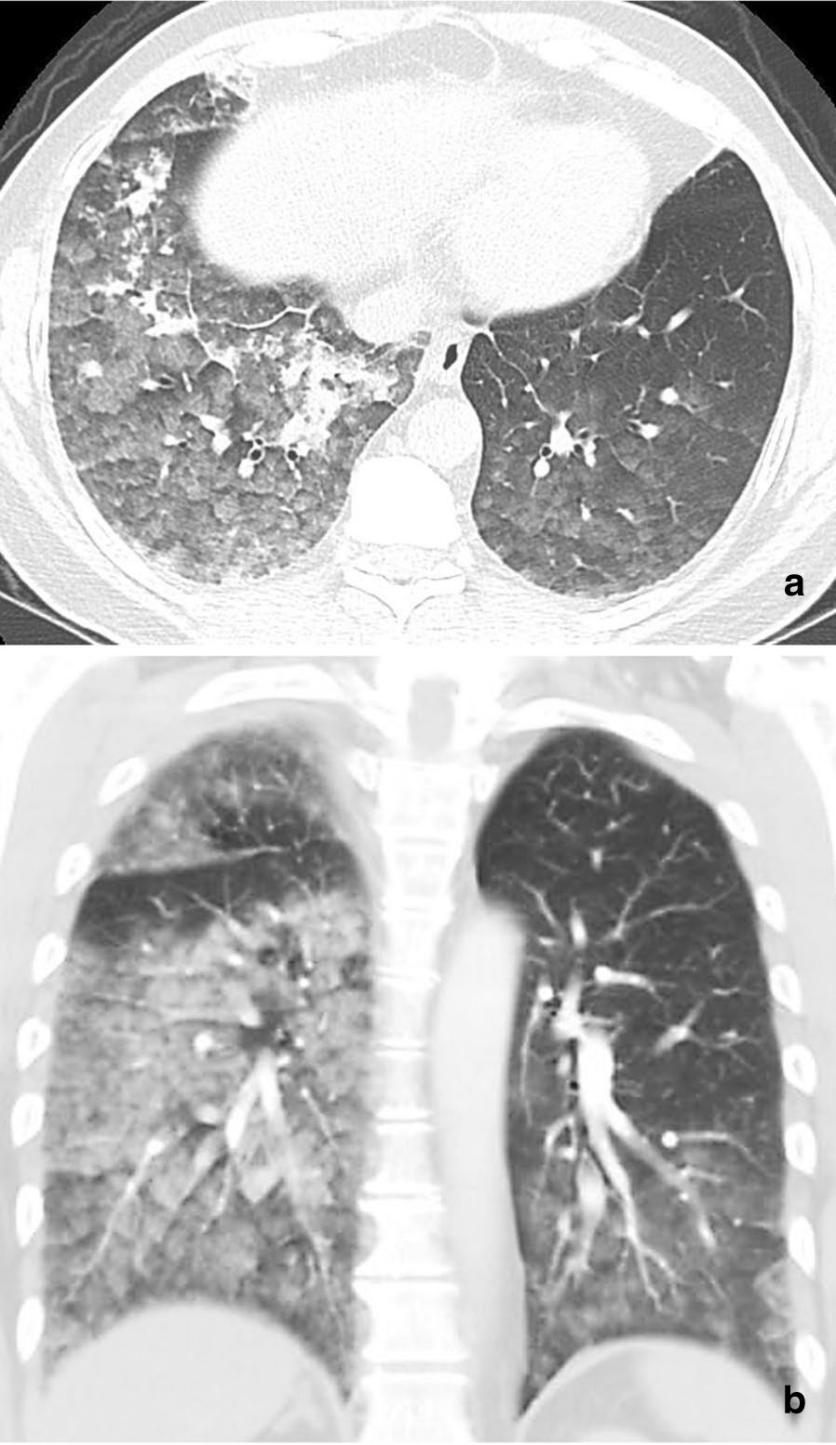
Non-infectious mimickers of indeterminate COVID-19. A 63-year-old man with diffuse alveolar hemorrhage secondary to thrombolytic therapy for myocardial infarction. **a**, **b** axial and coronal reformatted HRCT images show asymmetric bilateral patchy areas of GGO with small areas of consolidation

DAH is a severe and potentially fatal condition. Clinical history is important in diagnosis of DAH and differentiation from COVID-19. The presence of anemia, thrombocytopenia, coagulopathy, hemoptysis, collagen-vascular disease, pulmonary-renal syndromes (Wegener granulomatosis and Goodpasture syndrome) or recent bone marrow transplantation raises the possibility of DAH [47]**.** On CT, DAH often shows diffuse, patchy or lobular GGO and consolidation with perihilar and dependent distribution. A “crazy-paving” pattern occurs resulting from associated interlobular septal thickening (Fig. 11). DAH usually resolves within 10 days to 2 weeks [42, 47].

### Pulmonary alveolar proteinosis (PAP)

**Fig. 12:**
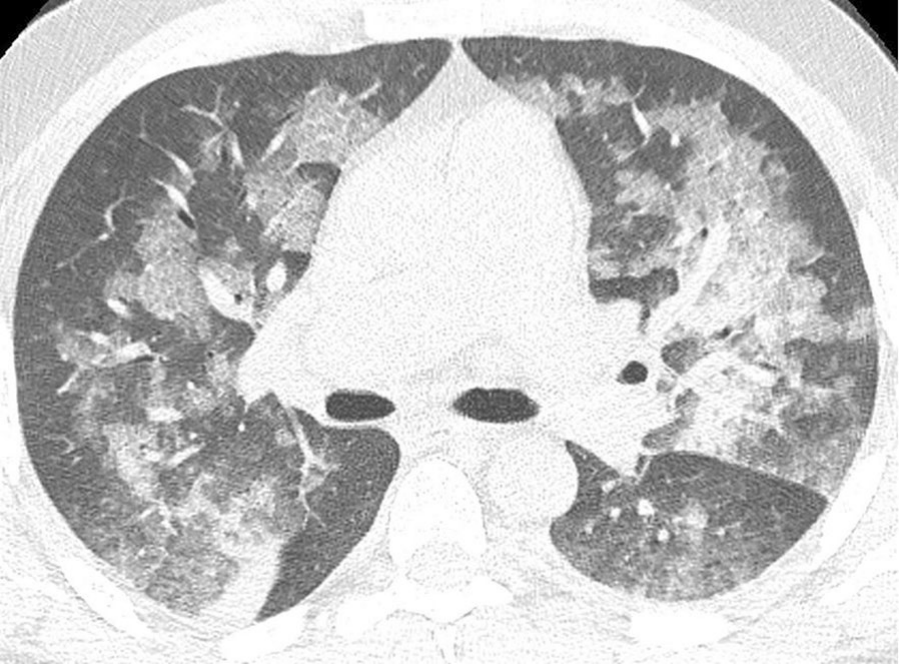
Non-infectious mimickers of indeterminate COVID-19. Pulmonary alveolar proteinosis proved by bronchoalveolar lavage (BAL) in a 38-year-old male smoker, axial CT image shows asymmetric bilateral patchy areas of GGO superimposed with interlobular septal thickening and crazy paving pattern, right-side fissural effusion is also noted

PAP is a rare lung disorder with three clinical forms: autoimmune, secondary and congenital. It is characterized by periodic acid Schiff stain positive material within the alveoli. CT shows areas of “crazy-paving” pattern that is more common in patients with autoimmune PAP than in secondary PAP (Fig. 12). Subpleural sparing, geographic distribution and lower lung predominance were also more frequent in autoimmune PAP [48]. Air space disease with patchy areas of parenchymal abnormalities may also reproduce a COVID-19 appearance; however, perihilar distribution in PAP is a differentiating point. Pulmonary nodules, pleural effusion and lymphadenopathy are not a feature of PAP but if present, then a superimposed infection should be considered. Clinically, PAP is different from COVID-19 as patients often present with low-grade fever, progressive exertional dyspnea and cough; however, patients may experience respiratory failure and require mechanical ventilation, which is an unusual but recognized complication [49].

### Interstitial lung abnormality (ILA)

ILA is increasingly recognized, and evolving descriptive term often encompasses different nondependent pulmonary abnormalities detected on chest CT and affecting more than 5% of any lung zone. It is observed in patients with no prior or established history of interstitial lung disease, in 4%–9% of smokers and 2%–7% of nonsmokers. Clinically, ILA manifested by chronic cough and shortness of breath, reduced total lung capacity and reduced exercise capacity [50–52]. It often progresses to a subclinical or early phase of lung fibrosis. Several non-fibrotic and fibrotic CT patterns have been described as ILA, including ground-glass opacities with or without reticulation, mosaic attenuation, centrilobular nodularity, honeycombing nonemphysematous cysts and traction bronchiectasis [50].

## Clinical mimickers of COVID-19 with atypical CT chest features

**Fig. 13:**
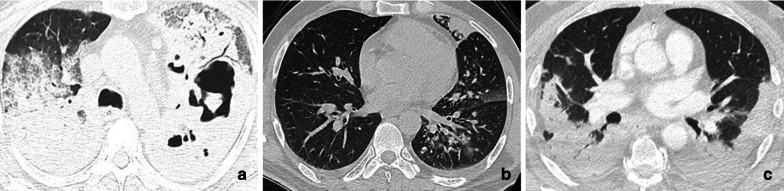
Atypical features for COVID-19. **a** A 47-year-old man with respiratory syncytial virus (RSV) pneumonia, axial HRCT image shows left pulmonary tree-in-bud opacities with centrilobular nodules, nearby patches of GGO and segmental consolidation anteriorly. **b** A 58-year-old woman with methicillin-resistant staphylococcus aureus (MRSA) pneumonia, HRCT shows bilateral consolidation with areas of GGO superimposed with crazy paving and cavitation on the left side. **c** A 67-year-old woman admitted to ICU with positive RT-PCR for COVID-19, axial HRCT show bilateral consolidation without significant GGO that was suggestive of coexisted bacterial infection. The causative organism of secondary infection was proved to be staphylococcus aureus, which is one of the commonest causes of hospital acquired infection

The clinical course of viral or bacterial pneumonia may simulate COVID-19; however, CT features such as perihilar predominance, centrilobular / tree in pud nodules, pleural effusion and lymphadenopathy are in favor of viral pneumonia rather than COVID-19. For example, RSV (respiratory syncytial virus) pneumonia was noted to be common in adults who require ICU admission in the winter season; unlike COVID-19, it shows an airway centric distribution, with bronchial wall thickening and tree in bud opacities, consolidation may or may not occur [53]. Varicella-zoster, measles and mumps typically present with multifocal centrilobular nodular infiltration and less commonly GGO (Fig. 13a) [23, 54]. In contrary to COVID-19, hilar lymphadenopathy and pleural effusion are common association with measles. Segmental or lobar consolidation with cavitation is a sign of bacterial pneumonia (Fig. 13b) or coexistence infection with COVID-19 (Fig. 13c). This common radiological appearance mostly cannot be used to predict the causative organism [53].

## Conclusion

A broad spectrum of pulmonary conditions demonstrates imaging features that mimic those of COVID-19 and are difficult to differentiate from it. Awareness of these conditions, careful radiologic analysis and attention to the clinical data are required to prevent an erroneous diagnosis that could potentially adversely impact management and patients’ outcome. A correct diagnosis of these conditions may prevent unnecessary hospitalization and reduce strict quarantine measures for all suspected patients that hold significant pressure on healthcare providers and medical infrastructure.

## Supplementary information

**Additional file 1**. Three-dimensional semitransparent volume-rendered reconstructions show the peripheral and basal distribution of the ground-glass opacities across both lungs for 54 years old man with COVID-19 positive RT-PCR test.

## Data Availability

All data generated during this study are included in this published article.

## Abbreviations

AFOP: Acute fibrinous organizing pneumonia
AIP: Acute interstitial pneumonia
ARDS: Acute respiratory distress syndrome.
COP: Cryptogenic organizing pneumonia
COVID-19: Coronavirus disease 2019
DAD: Diffuse alveolar damage
GGO: Ground-glass opacity
HRCT: High-resolution computed tomography
ICU: Intensive care unit
LDH: Lactate dehydrogenase
MERS: Middle East respiratory syndrome
OP: Organizing pneumonia
PJP: Pneumocystis jiroveci pneumonia
RT-PCR: Reverse transcription-polymerase chain reaction
SARS: Severe acute respiratory syndrome
SARS-CoV-2: Severe acute respiratory syndrome coronavirus 2
